# Reproducible Analysis of Rat Brain PET Studies Using an Additional [^18^F]NaF Scan and an MR-Based ROI Template

**DOI:** 10.1155/2012/580717

**Published:** 2012-09-23

**Authors:** Hans J. C. Buiter, Floris H. P. van Velden, Josée E. Leysen, Abraham Fisher, Albert D. Windhorst, Adriaan A. Lammertsma, Marc C. Huisman

**Affiliations:** ^1^Department of Nuclear Medicine & PET Research, VU University Medical Center, P.O. Box 7057, 1007 MB Amsterdam, The Netherlands; ^2^Israel Institute for Biological Research, P.O. Box 19, Ness-Ziona 74100, Israel

## Abstract

*Background*. An important step in the analysis of positron emission tomography (PET) studies of the brain is the definition of regions of interest (ROI). Image coregistration, ROI analysis, and quantification of brain PET data in small animals can be observer dependent. The purpose of this study was to investigate the feasibility of ROI analysis based on a standard MR template and an additional [^18^F]NaF scan. *Methods*. [^18^F]NaF scans of 10 Wistar rats were coregistered with a standard MR template by 3 observers and derived transformation matrices were applied to corresponding [^11^C]AF150(S) images. Uptake measures were derived for several brain regions delineated using the MR template. Overall agreement between the 3 observers was assessed by interclass correlation coefficients (ICC) of uptake data. In addition, [^11^C]AF150(S) ROI data were compared with *ex vivo* biodistribution data. *Results*. For all brain regions, ICC analysis showed excellent agreement between observers. Reproducibility, estimated by calculation of standard deviation of the between-observer differences, was demonstrated by an average of 17% expressed as coefficient of variation. Uptake of [^11^C]AF150(S) derived from ROI analysis closely matched *ex vivo* biodistribution data. *Conclusions*. The proposed method provides a reproducible and tracer-independent method for ROI analysis of rat brain PET data.

## 1. Introduction

An important step in the analysis of positron emission tomography (PET) data is the definition of regions of interest (ROI). For brain studies in man and large mammals, like primates and pigs, it becomes more common practice to use magnetic-resonance- (MR-) based templates [1–4]. For brain studies in small animals, for example, rats, several methods to define ROI have been proposed, for example, spatial normalization to an MR brain atlas [5], predefined PET templates [6, 7], direct ROI definition on PET images [8], ROI definition using a coregistered segmented rat brain atlas (based on autoradiography) [9], and probabilistic atlases based on PET data (e.g., [^18^F]FDG, [^18^F]FECT, and [^11^C]raclopride) for voxel-based functional mapping [10].

In case of small animals, automated image coregistration of PET data with MR templates is difficult, because of the relatively large difference in spatial resolution between MR and PET. In this study, small animal imaging was performed on a high-resolution PET scanner (i.e., ECAT high-resolution research tomograph, HRRT) with a spatial resolution of 2.5 to 3 mm and an MR scanner with a spatial resolution of 0.1 mm, resulting in an MR to PET difference of at least 25 in one direction, and 25^3^ in all directions. Furthermore, MR-PET coregistration techniques require a certain level of morphological correspondence between both images, that is, there should be sufficient mutual information in both types of images to allow automatic coregistration. Consequently, characterization of new PET tracers with highly specific uptake patterns can hamper direct coregistration of an MR template to a PET image, either manually or automatically.

A possible way to obtain anatomical information is to perform an additional [^18^F]NaF scan, which can be acquired immediately following an experimental PET study and which can then be used for coregistration to the MR template. As experiments are performed under anaesthesia and head fixation device, an implicit registration between both PET data sets can be assumed. [^18^F]NaF scans provide anatomical (skull) information, as uptake of [^18^F]NaF in bone is high with much lower uptake in brain tissue [11–13]. The outline of the skull in an [^18^F]NaF scan provides an image that, indirectly, is also available from an MR scan, where the skull can be visualized by inverting the image colour scale and setting this scale to maximum intensity. This provides a two-step method for MR-based ROI analysis of PET images in rat brains. The purpose of the present study was to investigate the reproducibility of MR-based ROI analysis using a standard MR template, coregistered with an additionally acquired [^18^F]NaF scan. This method was tested using [^11^C]AF150(S) in the rat brain [14]. [^11^C]AF150(S) is a functionally selective agonist radioligand with moderate affinity for the muscarinic acetylcholine receptor M1 (M1ACh-R) *in vitro* and is currently evaluated as a potential PET ligand for central nerve system imaging.

## 2. Methods

### 2.1. Animals

PET experiments were performed using 10 male Wistar rats (274 ± 24 g; Harlan Netherlands B.V., Horst, The Netherlands), who were kept in conditioned housing under a regular light/dark cycle (12/12 h), allowing food and water ad libitum. All animal experiments were performed in compliance with Dutch laws and were approved by the University Animal Ethics Committee (VU University Amsterdam, The Netherlands).

### 2.2. Tracer Synthesis

 [^18^F]NaF was prepared by passing an [^18^F]fluoride solution in water over a Waters quaternary methyl ammonium (QMA) light anion exchange cartridge (Waters Chromatography B.V., Etten-Leur, The Netherlands), followed by elution from the QMA cartridge with 1 mL 1.4% (w/v) of NaHCO_3_ in sterile H_2_O. This solution was collected in 10 mL of saline containing 7.09 mM NaH_2_PO_4_. Subsequently, the whole mixture was passed over a sterile Millex GV 0.22 *μ*m filter (Millipore B.V., Amsterdam, The Netherlands), yielding a sterile and pyrogen-free solution with radiochemical purity >99.9% and a pH of 7.

Quality control of the [^18^F]NaF solution was performed using an analytical high-performance liquid chromatography (HPLC) system consisting of a Jasco PU-2080 HPLC pump (Jasco, Ishikawa-cho, Japan), a Rheodyne 7724I injector (IDEX Health & Science, Wertheim-Mondfeld, Germany) with a 20 *μ*L loop, a Bio-Rad Aminex Fermentation Monitoring Column 150 × 7.8 mm (Bio-Rad, Hercules, CA, USA) with a Jasco UV-2075 Plus UV detector (Jasco, Ishikawa-cho, Japan) and a NaI radioactivity detector (Raytest, Straubenhardt, Germany). The purity of the product was determined using this analytical HPLC system eluted with water containing 0.001 M sulphuric acid at a flow rate of 0.8 mL·min^−1^.

[^11^C]AF150(S) was prepared as described in [14]. Briefly, [^11^C]AF150(S) was obtained via methylation of AF400 with [^11^C]CH_3_I. An incorporation yield of 90% of [^11^C]CH_3_ was achieved in a 5-minute reaction time at 60°C in CH_3_CN. The product was purified by HPLC, recovered by solid phase extraction and subsequently passed over a sterile Millex GV 0.22 *μ*m filter, yielding an isotonic, sterile, and pyrogen-free solution. The final solution of [^11^C]AF150(S) was obtained with a decay corrected overall yield of 69 ± 12% (*n* = 26), corresponding to 3200–7000 MBq at the end of the synthesis (i.e., 50 ± 5 min). Radiochemical purity was >99%, and specific activity at the end of synthesis was 23–118 GBq·*μ*mol^−1^.

### 2.3. PET Data Acquisition

PET measurements were performed using an ECAT HRRT (CTI/Siemens, Knoxville, TN, USA). The ECAT HRRT is a dedicated human brain PET scanner, with design features that enable high spatial resolution combined with high sensitivity, making it also suitable for small animal imaging. This scanner has a transaxial field of view of 312 mm and an axial field of view of 250 mm. The spatial resolution ranges from 2.3 to 3.2 mm full width at half maximum (FWHM) in transaxial direction and from 2.5 to 3.4 mm FWHM in axial direction [15]. 

First a transmission scan was performed using a 740 MBq 2D fan-collimated ^137^Cs rotating point source. Following this transmission scan, a 3D emission acquisition of 45 min was initiated immediately after an intravenous tail vein injection of 9.9 ± 0.6 MBq [^11^C]AF150(S). After the [^11^C]AF150(S) scan, an intravenous dose of 14.2 ± 3.5 MBq [^18^F]NaF was administered, followed 30 min later by a 3D emission of 30 min. Acquired data were stored in 64-bit list mode format and, for [^11^C]AF150(S), subsequently histogrammed into 21 time frames (7 × 10, 1 × 20, 2 × 30, 2 × 60, 2 × 150, and 7 × 300 s). [^18^F]NaF data were rebinned in a single frame of 30 min. Data were reconstructed using 3D ordered subsets weighted least squares (3D-OSWLS) [16] using 7 iterations and 16 subsets. All data were normalized and corrected for attenuation, randoms, scatter, decay, and dead-time. All images were reconstructed into a matrix of 256 × 256 × 207 voxels with a voxel size of 1.218 × 1.218 × 1.218 mm^3^.

### 2.4. MR Template

MR data were provided by the Image Sciences Institute of the University Medical Center Utrecht and consisted of a typical Wistar rat brain image, obtained by averaging single MR images of 8 Wistar rat brains (mean weight 305 g) acquired on a 4.7 Tesla MR system (Varian, Palo Alto, CA, USA) using a 3D Fast Spin Echo sequence (TR/TE = 1500/10 ms, 128 × 128 × 128 voxels, 32 × 40 × 32 mm^3^). Final image dimensions were 144 × 112 × 152 voxels with voxel dimensions of 0.125 × 0.125 × 0.156 mm^3^.

Voxel dimensions of the MR image were scaled to 0.1 × 0.1 × 0.13 mm^3^, as brain size did not correspond with that in the present study. As an index of brain size, the distance between left and right dorsal lateral sides of the tympanic bulla was used. This distance was 14.4 mm in the original average MR image and 11.4 ± 0.1 mm for rats used in the present study (as was calculated from the [^18^F]NaF images).

The MR template was generated by defining ROI on this average MR image using AMIDE (version 0.9.1; http://amide.sourceforge.net; [17]). ROI was defined from the beginning of the striatum until the end of the medulla oblongata, corresponding to plates 13 to 130 from the Paxinos and Watson atlas of the rat brain [18].

### 2.5. MR Template Coregistration

**Figure 1:**
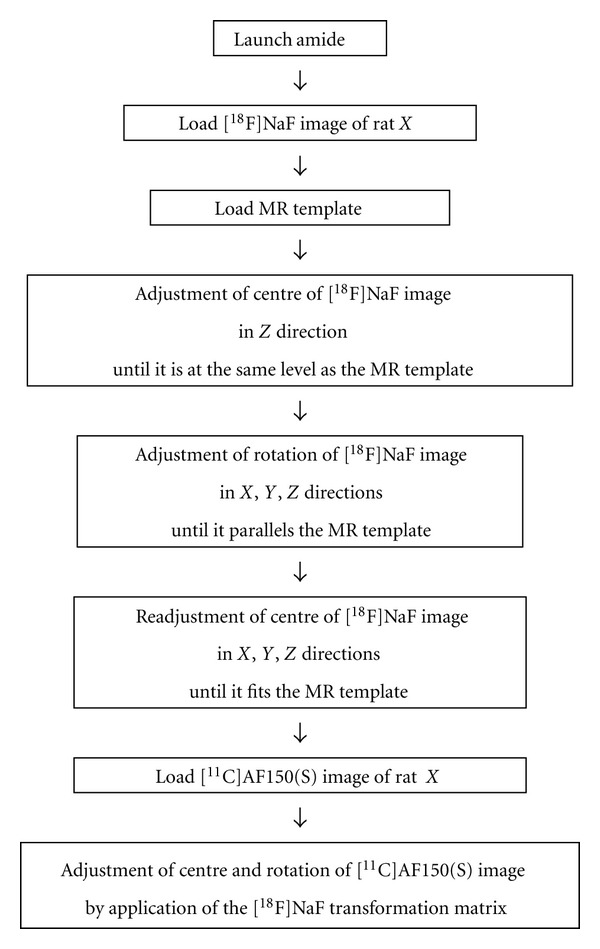
Overview of the various steps to manually coregister [^18^F]NaF and [^11^C]AF150(S) images with the MR template.

**Figure 2:**
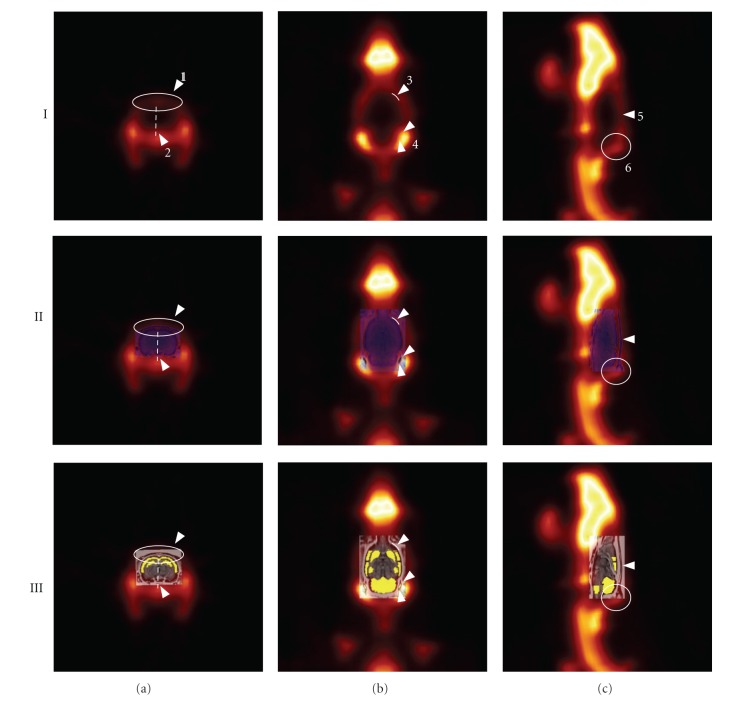
Example of an [^18^F]NaF image coregistered with MR image and the MR template. (I) [^18^F]NaF image, coregistered with (II) MR image and (III) MR template for (a) coronal, (b) transverse, and (c) sagittal cross-sections. Arrows indicate (1) medial surface cranial case, (2) ipsilateral line distributing hemispheres, (3) frontal bones, (4) tympanic bulla, (5) surface of cranial case, and (6) articulation of parietal and occipital bones.

Using AMIDE, the [^18^F]NaF image of each individual animal was coregistered manually with the MR template image by three different observers according to a predefined protocol. An overview of the steps involved in this manual image coregistration is depicted in Figure 1. Briefly, the centre of an [^18^F]NaF image was moved until it roughly overlaid the MR template. Next, rotational settings were adjusted until the [^18^F]NaF image paralleled the MR template, and, finally, the centre was adjusted again until the image fitted the MR template. MR template and [^18^F]NaF image fits were considered successful when (a) the medial surface of the cranial case was aligned and the hemispheres were distributed ipsilaterally in a coronal cross-section, (b) tympanic bullae and frontal bones of the anterior part of the cranial cavity were aligned in a transverse cross-section, and (c) the surface of the cranial case and the articulation of parietal and occipital bones were aligned in a sagittal cross-section, as illustrated in Figure 2. Finally, dynamic [^11^C]AF150(S) data were coregistered by directly applying the (rotational) settings obtained from the corresponding [^18^F]NaF coregistration.

### 2.6. ROI Analysis

Validation of ROI analysis software was performed on simulated data using AMIDE. ROI of the MR template was projected onto two simulated PET images that consisted of voxels with a theoretical content of either 0 or 1 kBq, arranged in a 3D checkerboard pattern. One image had voxel dimensions equal to the MR image, whereas the other had voxel dimensions equal to the PET image. Since there is a considerable difference in spatial resolution with a factor of 1390 difference in voxel volumes, the purpose of this simulation was to check whether ROI volumes and mean ROI activity were similar for both images.

For ROI analysis of experimental data, the MR template was projected onto the coregistered [^11^C]AF150(S) images, using the corresponding [^18^F]NaF image. The percentage injected dose per gram tissue (%ID/g) was calculated for each brain region using the following equation:

(1) %IDg  =regional  radioactivity  concentration  (kBq/mL)(injected  dose  (kBq)×density  brain  tissue  (g/mL))   ×100%

in which a mean brain tissue density of 1.045 g/mL was used [19].

### 2.7. Evaluation of Image Coregistration

Interobserver variability of the image coregistration method was investigated by comparing results of the three independent observers for all brain regions specified. The reproducibility of image coregistration was assessed by considering the variability within subjects not attributable to possible systematic differences between observers. It was assumed that the within-subject variability was the same for all subjects when there were no statistically significant differences, and it was estimated by calculating the standard deviation of the between-observer differences recorded for each subject. Expressed as a coefficient of variation (COV), this standard deviation provides a dimensionless measure of reproducibility. Reliability between the 3 observers was quantified using the intraclass correlation coefficient for absolute agreement (ICC_A_) and consistency (ICC_C_). Validity of the image coregistration method was verified comparing PET derived %ID/g data with previously obtained measurements of %ID/g in *ex vivo* biodistribution studies. Briefly, 5 groups of 4 of rats received an intravenous injection of [^11^C]AF150(S) and were sacrificed at 5, 10, 15, 30, or 60 min after injection. Brains were excised and regions dissected. All brain regions were weighed and counted for radioactivity using an automatic gamma counter (Wallac 1282 Compugamma CS, LKB Wallac, Turku, Finland), and the %ID/g was calculated as percentage of total radioactivity injected, divided by the weight of the tissue.

### 2.8. Statistics

All statistical analyses were performed using SPSS Statistics (version 17.0; SPSS Inc., Chicago, IL, USA). The Shapiro-Wilk test was used to confirm that all data were normally distributed. A two-tailed paired Student's *t*-test was used for ROI analysis of the checkerboard simulation study. The correlation between ROI results (ROI volume and mean ROI activity concentration) of MR and PET checkerboard images was assessed using squared Pearson's correlation. A two-way ANOVA with Bonferroni posttest was used for comparison of %ID/g measures obtained by the various observers who delineated ROI for [^11^C]AF150(S) PET data and for the comparison of PET and *ex vivo* derived %ID/g values. The interobserver reliability for [^11^C]AF150(S) PET data (mean ROI %ID/g) was computed by intraclass correlation coefficient using a two-way mixed effects model for absolute agreement (ICC_A_) and consistency (ICC_C_). ICC scores range from 0 to 1, representing a level of agreement: ≤0.40 poor to fair, 0.41–0.60 moderate, 0.61–0.80 substantial, 0.81–1.00 almost perfect [20]. Confidence intervals were also calculated for each ICC score. Data are reported as mean ± SD, and differences were considered significant at a level of *P* < 0.05.

## 3. Results

### 3.1. MR Template

Table 1 provides an overview of the ROI defined on the MR image.

### 3.2. ROI Analysis

**Figure 3:**
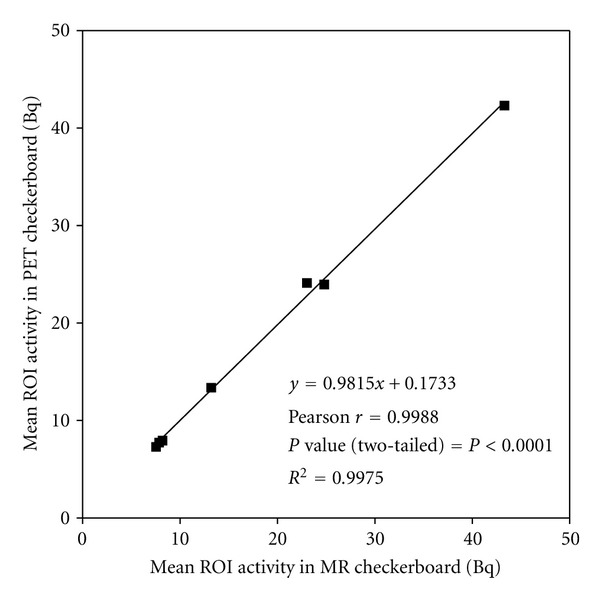
Pearson's correlation plot of mean ROI activity for all brain regions in PET versus MR checkerboard.

Both ROI volumes and mean ROI activity concentrations in left and right striata, posterior cortex, and medulla oblongata were slightly lower for the PET than for the MR resolution checkerboard images. The opposite was true for hippocampus, frontal cortex, and cerebellum, as shown in Table 2. More importantly, both ROI volumes and mean ROI activities derived from MR and PET checkerboard images were not significantly different from each other. Correlation analysis of mean ROI activity concentrations in MR and PET resolution checkerboard images yielded a slope of 0.982, an intercept of 0.173, and an *R* ^2^ of 0.998 (Figure 3).

### 3.3. Evaluation of Image Coregistration

Quantitative reproducibility of image coregistration was assessed using [^11^C]AF150(S) data by comparing %ID/g data between observers at five different time points (Table 3). The Shapiro-Wilk test confirmed that these data were consistent with the normal distribution (*W* statistic ≥ 0.936, *P* ≥ 0.511). The comparison of %ID/g data revealed that for each time point none of the observations were significantly different from each other (*P* > 0.722). In addition, COV values were not significantly different between the 3 observers (*P* > 0.467).

Interobserver reliability of the image coregistration method was assessed by calculating ICC_A_ and ICC_C_ of mean uptake in each region, as derived by the 3 observers. These results are summarized in Table 4 and, for all regions, show almost perfect agreement (ICC_A_ > 0.856) between observers. In addition, for all regions, there was almost perfect consistency (ICC_C_ > 0.939) between observers.

**Figure 5:**
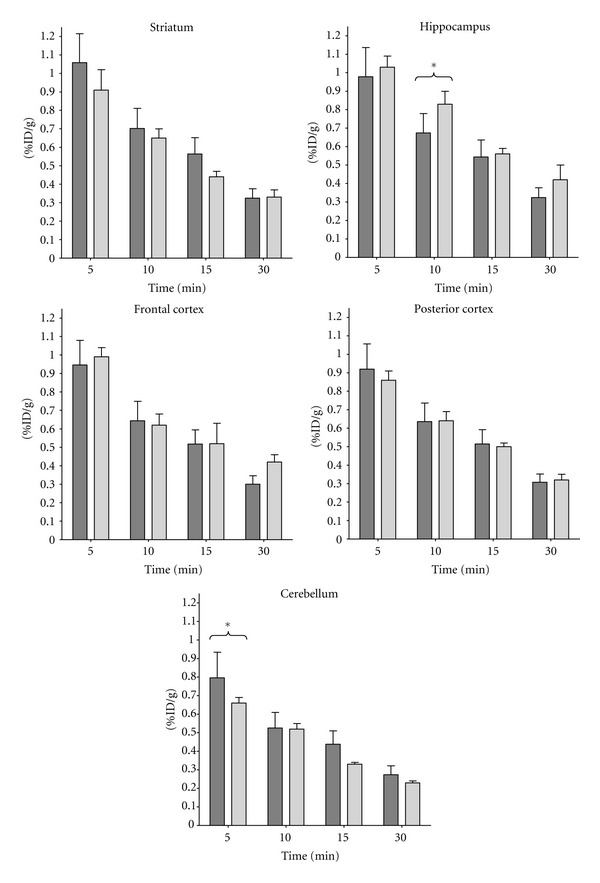
Uptake (%ID/g) of [^11^C]AF150(S) in various brain regions obtained from PET and *ex vivo* biodistribution experiments. PET studies, dark gray (*n* = 10, average of 3 observers), and *ex vivo* biodistribution experiments, light gray (*n* = 4 per time point). Data are presented as mean ± SD. Level of significance was evaluated using a two-way ANOVA with Bonferroni posttest; *P < 0.05.

Observed regional brain uptake of [^11^C]AF150(S) was compared between PET and *ex vivo* studies. The Shapiro-Wilk test (*W* statistic ≥ 0.885, *P* ≥ 0.361) confirmed that these data were consistent with the normal distribution. A graphical overview for the various time points is given in Figure 5, indicating no significant differences between both datasets at any of the time points, except for cerebellum at 5 min and hippocampus at 10 min.

## 4. Discussion

### 4.1. Data Acquisition

The proposed generic method for ROI analysis of PET data acquired in rats is based on the acquisition of an additional [^18^F]NaF scan. In the present study, the additional acquisition time was 30 min, starting 30 min after injection of ~14 MBq of [^18^F]NaF. This time frame, however, can be adapted according to experimental design in future studies.

### 4.2. ROI Analysis

In order to determine effects of different spatial resolutions (1390 MR voxels correspond with one PET voxel) on ROI analyses, calculation of average ROI values and volumes was validated using a simulated checkerboard scan. In general, less than 7% difference in volumes and less than 5% in mean ROI activity values were observed between PET images with PET and MR resolution (Table 2) and none of these differences were significant. In addition, mean ROI activity values derived from both images showed near perfect correlation (*R* ^2^ > 0.998; slope = 0.982). Therefore, applying MR-based ROI onto PET images is reproducible with respect to both ROI volumes and mean ROI activity values.

### 4.3. Evaluation of the Image Coregistration Paradigm

All three observers successfully coregistered all ten [^18^F]NaF images with the MR brain template, following the procedure as outlined in Figure 1. Derived uptake of [^11^C]AF150(S) was not significantly different between observers for any brain region and any time point. Following image coregistration of [^11^C]AF150(S) scans, interobserver agreement (ICC_A_ and ICC_C_) of uptake was excellent for all brain regions. This implies that the image coregistration procedure is very reliable. Moreover, uptake values were similar to those obtained from an *ex vivo* study. The minor differences at 10 min for hippocampus and at 5 min for cerebellum might be ascribed to individual variability in combination with the relatively small groups of animals. Interestingly, both PET and *ex vivo* biodistribution results indicate that [^11^C]AF150(S) uptake is highest in striatum, followed by hippocampus, then cortex and then cerebellum, closely following the distribution of M1ACh-R in rat brain [21].

Clearly, some user-dependent uncertainty may remain in the coregistration of MR template and [^18^F]NaF images. Based on the good interobserver correspondence in the manual procedure, semiautomatic or fully automatic image coregistration methods could be investigated for easier and faster ROI analysis of small animal PET studies.

 Although several methods are available for PET coregistration in small animals [5–7, 9, 10], reproducibility was not assessed for all those methods. In two previous small animal studies reproducibility of brain uptake has been investigated [8, 22] using direct ROI definition on PET images or predefined PET templates for [^11^C]raclopride scans. Reproducibility of [^11^C]raclopride uptake was found to be between 10% and 22%. These values are similar to the reproducibility of [^11^C]AF150(S) uptake observed in the present study (average COV of 17%), suggesting that there is no additional uncertainty due to application of the presently described two-step method of ROI analysis. The advantage of the present method is that it allows exact localization of the brain, independent of radioligand and PET scanner used, and without the need to perform an individual MR scan for each rat.

 In the near future, multimodality imaging may provide better means for analysis of small animal PET studies. The presently available combined PET/CT small animal scanners do have certain notable shortcomings, that is, the inability to perform simultaneous data acquisition and lesser contrast among soft tissues compared to MR. Nevertheless, the CT component of PET/CT images may be used to coregister these images with MR. Ultimately, the emerging role of hybrid PET/MR small animal imaging may provide better means for tracer-independent data analysis of rat brain studies and should be further investigated.

## 5. Conclusions

Reproducible analysis of PET data is possible using coregistration of an MR-based ROI template with an additional [^18^F]NaF scan. This method can serve as a reliable and reproducible two-step method for tracer-independent data analysis of rat brain studies, as demonstrated for [^11^C]AF150(S) data.

## Figures and Tables

**Figure 4:**
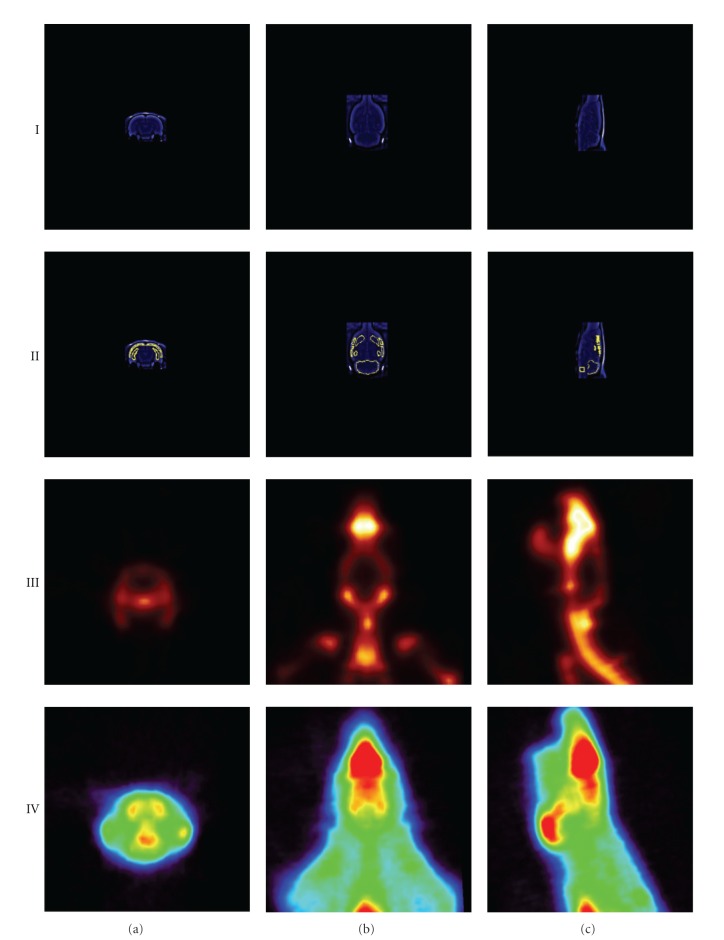
Overview of coregistered MR image and template with PET images. (I) MR image, (II) MR template, (III) [^18^F]NaF image, and (IV) [^11^C]AF150(S) image (0–45 min) for (a) coronal, (b) transverse, and (c) sagittal cross-sections.

**Table 1:** Regions and volumes of ROI defined on the MR template.

Brain region	Volume (mm^3^)
Left striatum	16.4
Right striatum	15.1
Hippocampus	26.4
Frontal cortex (including part parietal cortex)	46.1
Posterior cortex (including occipital cortex)	49.6
Medulla oblongata (part)	15.7
Cerebellum	86.6

**Table 2:** Volume, mean activity concentration, and total activity in various ROIs projected onto the checkerboard images with MR and PET resolution.

Brain region	MR image			PET image		
	Volume (mm^3^)	Mean voxel Activity concentration^a^ (kBq·mL^−1^)	Mean ROI activity (Bq)	Volume (mm^3^)	Mean voxel Activity concentration^b^ (kBq·mL^−1^)	Mean ROI activity (Bq)
Left striatum	16.4	0.50	8.19	15.3	0.52	7.95
Right striatum	15.1	0.50	7.56	14.7	0.50	7.30
Hippocampus	26.4	0.50	13.22	26.8	0.50	13.38
Frontal cortex	46.1	0.50	23.03	48.6	0.50	24.13
Posterior cortex	49.6	0.50	24.80	48.9	0.49	23.96
Medulla oblongata	15.7	0.50	7.85	15.8	0.49	7.75
Cerebellum	86.7	0.50	43.35	86.0	0.49	42.14

^a^1 MR voxel = 0.0013 mm^3^.

^b^1 PET voxel = 1.81 mm^3^.

**Table 3:** [^11^C]AF150(S) uptake (%ID/g) derived from various brain regions by three observers following image coregistration.

Number of subjects (*n* = 10)								
	Region							
	Left striatum	Right striatum	Hippocampus	Frontal cortex	Posterior cortex	Medulla oblongata	Cerebellum	Mean COV
Observer 1								
5 min	1.06 ± 0.16	1.04 ± 0.17	0.98 ± 0.16	0.95 ± 0.13	0.92 ± 0.14	0.67 ± 0.13	0.80 ± 0.14	16.12
10 min	0.70 ± 0.11	0.71 ± 0.11	0.67 ± 0.10	0.64 ± 0.11	0.64 ± 0.10	0.46 ± 0.08	0.53 ± 0.08	16.08
15 min	0.57 ± 0.09	0.57 ± 0.10	0.54 ± 0.09	0.52 ± 0.08	0.52 ± 0.08	0.38 ± 0.06	0.44 ± 0.07	15.96
30 min	0.33 ± 0.05	0.33 ± 0.05	0.32 ± 0.05	0.30 ± 0.05	0.31 ± 0.05	0.25 ± 0.04	0.27 ± 0.05	15.81
45 min	0.23 ± 0.04	0.23 ± 0.05	0.23 ± 0.04	0.21 ± 0.04	0.22 ± 0.04	0.18 ± 0.04	0.20 ± 0.04	18.49
Observer 2								
5 min	1.05 ± 0.17	1.05 ± 0.18	0.99 ± 0.16	0.99 ± 0.17	0.95 ± 0.17	0.66 ± 0.12	0.80 ± 0.14	17.26
10 min	0.70 ± 0.12	0.72 ± 0.12	0.68 ± 0.11	0.67 ± 0.13	0.65 ± 0.12	0.47 ± 0.08	0.53 ± 0.09	17.19
15 min	0.57 ± 0.10	0.58 ± 0.10	0.55 ± 0.09	0.54 ± 0.10	0.53 ± 0.09	0.39 ± 0.06	0.44 ± 0.08	17.10
30 min	0.33 ± 0.05	0.33 ± 0.05	0.33 ± 0.05	0.31 ± 0.05	0.31 ± 0.05	0.24 ± 0.04	0.28 ± 0.05	16.67
45 min	0.23 ± 0.04	0.24 ± 0.04	0.23 ± 0.04	0.22 ± 0.04	0.22 ± 0.04	0.18 ± 0.03	0.20 ± 0.04	18.08
Observer 3								
5 min	1.06 ± 0.16	1.04 ± 0.17	0.98 ± 0.15	0.93 ± 0.14	0.89 ± 0.14	0.71 ± 0.14	0.80 ± 0.14	16.44
10 min	0.70 ± 0.12	0.71 ± 0.12	0.67 ± 0.11	0.64 ± 0.11	0.61 ± 0.10	0.48 ± 0.07	0.53 ± 0.09	16.52
15 min	0.56 ± 0.09	0.57 ± 0.10	0.54 ± 0.09	0.51 ± 0.08	0.50 ± 0.08	0.40 ± 0.06	0.44 ± 0.07	16.51
30 min	0.32 ± 0.06	0.33 ± 0.05	0.32 ± 0.05	0.30 ± 0.05	0.29 ± 0.05	0.25 ± 0.04	0.28 ± 0.05	16.70
45 min	0.22 ± 0.04	0.23 ± 0.04	0.23 ± 0.04	0.21 ± 0.04	0.21 ± 0.04	0.19 ± 0.04	0.20 ± 0.04	18.86

Data represent mean ± SD (*n* = 10).

**Table 4:** Intraclass correlation coefficients and corresponding 95% confidence intervals for regional [^11^C]AF150(S) brain uptake (%ID/g).

Number of subjects (*n* = 10)								
ROI	Observer 1 versus 2				Observer 1 versus 3			
	ICC_A_	95% CL	ICC_C_	95% CL	ICC_A_	95% CL	ICC_C_	95% CL
Left striatum	0.998	(0.991, 0.999)	0.997	(0.990, 0.999)	0.997	(0.988, 0.999)	0.997	(0.988, 0.999)
Right striatum	0.995	(0.978, 0.999)	0.995	(0.981, 0.999)	0.998	(0.991, 0.999)	0.998	(0.992, 0.999)
Hippocampus	0.997	(0.989, 0.999)	0.997	(0.989, 0.999)	0.992	(0.769, 0.999)	0.997	(0.989, 0.999)
Frontal cortex	0.930	(0.491, 0.985)	0.961	(0.853, 0.990)	0.985	(0.886, 0.997)	0.991	(0.963, 0.998)
Posterior cortex	0.956	(0.826, 0.989)	0.962	(0.856, 0.990)	0.927	(0.102, 0.987)	0.978	(0.913, 0.994)
Medulla oblongata	0.966	(0.879, 0.991)	0.967	(0.873, 0.992)	0.904	(−0.022, 0.984)	0.985	(0.940, 0.996)
Cerebellum	0.993	(0.972, 0.998)	0.992	(0.969, 0.998)	0.970	(0.499, 0.994)	0.988	(0.954, 0.999)

	Observer 2 versus 3				Overall			
Left striatum	0.997	(0.988, 0.999)	0.997	(0.989, 0.999)	0.997	(0.992, 0.999)	0.997	(0.992, 0.999)
Right striatum	0.998	(0.991, 0.999)	0.997	(0.990, 0.999)	0.997	(0.990, 0.999)	0.997	(0.991, 0.999)
Hippocampus	0.987	(0.632, 0.998)	0.996	(0.983, 0.999)	0.992	(0.947, 0.998)	0.997	(0.991, 0.999)
Frontal cortex	0.907	(0.080, 0.983)	0.968	(0.878, 0.992)	0.937	(0.682, 0.985)	0.972	(0.922, 0.992)
Posterior cortex	0.856	(0.062, 0.971)	0.939	(0.776, 0.985)	0.911	(0.605, 0.979)	0.959	(0.885, 0.989)
Medulla oblongata	0.895	(0.306, 0.977)	0.945	(0.794, 0.986)	0.919	(0.608, 0.981)	0.966	(0.904, 0.991)
Cerebellum	0.957	(0.663, 0.991)	0.977	(0.910, 0.994)	0.974	(0.881, 0.994)	0.986	(0.960, 0.996)

Level of agreement: ≤0.40 poor to fair, 0.41–0.60 moderate, 0.61–0.80 substantial, 0.81–1.00 almost perfect.

## References

[1] Mazziotta JC, Toga AW, Evans A, Fox P, Lancaster J (1995). A probabilistic atlas of the human brain: theory and rationale for its development: the International Consortium for Brain Mapping (ICBM). *NeuroImage*.

[2] Black KJ, Koller JM, Snyder AZ, Perlmutter JS (2001). Template images for nonhuman primate neuroimaging: 2. Macaque. *NeuroImage*.

[3] Andersen F, Watanabe H, Bjarkam C, Danielsen EH, Cumming P (2005). Pig brain stereotaxic standard space: mapping of cerebral blood flow normative values and effect of MPTP-lesioning. *Brain Research Bulletin*.

[4] Svarer C, Madsen K, Hasselbalch SG (2005). MR-based automatic delineation of volumes of interest in human brain PET images using probability maps. *NeuroImage*.

[5] Fujita M, Zoghbi SS, Crescenzo MS (2005). Quantification of brain phosphodiesterase 4 in rat with (R)-[^11^C]Rolipram-PET. *NeuroImage*.

[6] Kuntner C, Kesner AL, Bauer M (2009). Limitations of small animal PET imaging with [^18^F]FDDNP and FDG for quantitative studies in a transgenic mouse model of alzheimer’s disease. *Molecular Imaging and Biology*.

[7] Doorduin J, Klein HC, de Jong JR, Dierckx RA, de Vries EFJ (2010). Evaluation of [^11^C]-DAA1106 for imaging and quantification of neuroinflammation in a rat model of herpes encephalitis. *Nuclear Medicine and Biology*.

[8] Alexoff DL, Vaska P, Marsteller D (2003). Reproducibility of ^11^C-raclopride binding in the rat brain measured with the microPET R4: effects of scatter correction and tracer specific activity. *Journal of Nuclear Medicine*.

[9] Topping GJ, Dinelle K, Kornelsen R, McCormick S, Holden JE, Sossi V (2010). Positron emission tomography kinetic modeling algorithms for small animal dopaminergic system imaging. *Synapse*.

[10] Casteels C, Vermaelen P, Nuyts J (2006). Construction and evaluation of multitracer small-animal PET probabilistic atlases for voxel-based functional mapping of the rat brain. *Journal of Nuclear Medicine*.

[11] Volker JF, Hodge HC, Wilson HJ (1940). The adsorption of fluorides by enamel, dentin, bone, and hydroxyapatite as shown by the radioactive isotope. *Journal of Biological Chemistry*.

[12] Posner AS, Eanes ED, Harper RA, Zipkin I (1963). X-ray diffraction analysis of the effect of fluoride on human bone apatite. *Archives of Oral Biology*.

[13] Scarpa M, Vianello F, Rigo A, Viglino P, Bracco F, Battistin L (1993). Uptake and life time of fluoride ion in rats by 19F-NMR. *Magnetic Resonance Imaging*.

[14] Buiter HJC, Leysen JE, Schuit RC (2012). Radiosynthesis and biological evaluation of the M1 muscarinic acetylcholine receptor agonist ligand [^11^C]AF150(S). *Journal of Labelled Compounds and Radiopharmaceuticals*.

[15] De Jong HWAM, Van Velden FHP, Kloet RW, Buijs FL, Boellaard R, Lammertsma AA (2007). Performance evaluation of the ECAT HRRT: an LSO-LYSO double layer high resolution, high sensitivity scanner. *Physics in Medicine and Biology*.

[16] Van Velden FHP, Kloet RW, Van Berckel BNM, Lammertsma AA, Boellaard R (2009). Accuracy of 3-dimensional reconstruction algorithms for the high-resolution research tomograph. *Journal of Nuclear Medicine*.

[17] Loening AM, Gambhir SS (2001). AMIDE: a completely free system for medical imaging data analysis. *The Journal of Nuclear Medicine*.

[18] Paxinos G, Watson C (2007). *The Rat Brain in Stereotaxic Coordinates*.

[19] DiResta GR, Lee J, Arbit E (1991). Measurement of brain tissue specific gravity using pycnometry. *Journal of Neuroscience Methods*.

[20] Landis JR, Koch GG (1977). The measurement of observer agreement for categorical data. *Biometrics*.

[21] Mash DC, Potter LT (1986). Autoradiographic localization of M1 and M2 muscarine receptors in the rat brain. *Neuroscience*.

[22] Hume SP, Lammertsma AA, Myers R (1996). The potential of high-resolution positron emission tomography to monitor striatal dopaminergic function in rat models of disease. *Journal of Neuroscience Methods*.

